# Uniform two-dimensional crystals of polystyrene nanospheres fabricated by a surfactant-assisted spin-coating method with polyoxyethylene tridecyl ether

**DOI:** 10.1038/s41598-019-47990-z

**Published:** 2019-08-07

**Authors:** Byoungchoo Park, Seo Yeong Na, In-Gon Bae

**Affiliations:** Department of Electrical and Biological Physics, Kwangwoon University, Wolgye-Dong, Nowon-gu, Seoul, 01897 Republic of Korea

**Keywords:** Nanoparticles, Photonic crystals, Photonic devices

## Abstract

Spin-coated self-assemblies of colloidal particles have been developed recently as an attractive close-packed monolayer of the particles for a variety of applications, but they are limited by the small area of their monolayers, especially given their low uniformity and monolayer coverage on large-area substrates. We report several noteworthy characteristics of a close-packed monolayer of polystyrene nanospheres (PS NSs) fabricated using a simple and inexpensive spin-coating method with a PS NS suspension mixed using the nonionic surfactant polyoxyethylene (12) tridecyl ether (PEO-TDE). In our study, we show that the PEO-TDE surfactant offers excellent wettability, surface tension, and a slow solvent evaporation rate of the PS NS suspension, similar to the conventional surfactant Triton X-100. We demonstrate that the relatively high monolayer coverage with reduced defects is produced when introducing the PEO-TDE surfactant. Specifically, monolayer coverage of more than 95% on a Si substrate was achieved, which is much better than that with the typical Triton X-100, and is one of the highest coverage rates realized by a spin-coating method. This excellent uniformity of the PS NS monolayer with high monolayer coverage is mainly attributed to the relatively low viscosity of the PS NS suspension, even at high concentrations of PEO-TDE. Moreover, the PEO-TDE surfactant provides highly uniform monolayers on a large-scale glass substrate even for large-sized PS NSs. We also highlight the fact that the PEO-TDE surfactant has another advantage in that the spin-coating process of the PS NS suspension can be done under common ambient laboratory conditions, unlike those required for the highly toxic Triton X-100. We therefore conclude that PEO-TDE can be a useful surfactant during the fabrication of close-packed monolayers for various applications owing to its simple and straightforward control of PS NSs, its uniform and high surface coverage, and due to the safety of the fabrication process.

## Introduction

High uniformity and monolayer coverage of a self-assembled monolayer over large areas are fundamental requirements for the deposition processes of colloidal particles for use with shadow nanosphere lithography^1–5^, 2D optical devices^6^, light-trapping in solar cells^7^, and bio/chemical sensors^8–11^. Owing to these various applications, several different techniques have been developed to fabricate uniform monolayer arrays of colloidal particles. Examples include the Langmuir-Blodgett deposition^12,13^, convective self-assembly^6,14,15^, dip-coating^16,17^, and slop self-assembly^18–20^ processes. Although these techniques have successfully provided close-packed monolayers of colloidal particles with high uniformity, the limited sizes of the monolayers and the slow deposition process impede their wider use. Due to these issues, simple and inexpensive spin-coating methods have been developed to offer large-area self-assembled monolayers of colloidal particles^1–5,21–24^. However, the poor surface wettability of a colloidal suspension and/or the rapid evaporation rates of common solvents used in these suspensions remain as considerable challenges to those who study and use spin-coating processes^1,21^. Hence, there is a strong need for well-designed colloidal suspensions both suitable for the spin-coating process and capable of providing a uniform close-packed monolayer of colloidal particles with high monolayer coverage.

Recently, to improve the solution wettability and to delay the evaporation rates of colloidal suspensions, additional surfactant mixtures were introduced for use in the suspensions^1,4,22,25^. For example, polyethylene glycol tert-octylphenyl ether (Polyoxyethylene octyl phenyl ether, Triton X-100) is a conventional surfactant used to improve the interparticle interaction and delayed evaporation rate of methanol-based suspensions containing colloidal particles of polystyrene nanosphere (PS NS)^4^. However, this approach using the Triton X-100 surfactant is still limited to a few suspensions given their high viscosity levels at high concentrations^26^, making it more difficult to produce a uniform monolayer of nanospheres even with the delayed solvent evaporation rate. Instead of Triton X-100, ethylene glycol has also been utilized as a surfactant, but its high viscosity also causes the issues which arise when using Triton X-100^25,27^. Therefore, it remains a considerable challenge to find an appropriate surfactant for the production of a highly uniform monolayer of nanospheres using the spin-coating approach.

In this article, we introduce a new surfactant of polyoxyethylene (12) tridecyl ether (PEO-TDE), having a covalently bonded oxygen-containing hydrophilic group and a hydrophobic alkyl chain structure, for improved monolayer formation of PS NSs on a Si surface. Accordingly, we observe that a uniform PS NS monolayer with reduced defects, such as voids and multilayers, can be produced by the spin-coating method with the help of the PEO-TDE surfactant. The quality of the PS NS monolayer fabricated with PEO-TDE is also compared to such a surface created with Triton X-100. From the comparison, it could be confirmed that the relatively low viscosity of the suspension at a high concentration of PEO-TDE plays an important role in the enhanced uniformity of the PS NS monolayer, even on a large-area coating. Considering the difficulty of controlling the properties of colloidal suspensions, our results reveal the major contribution of the PEO-TDE surfactant in PS NS suspensions, strongly suggesting the feasibility of this surfactant for use in a variety of devices, as mentioned above. The lower toxicity compared to that of Triton X-100 makes it an even more attractive choice.

## Results and Discussion

### Surface wettabilities of PS NS suspensions and their monolayer formations

**Figure 1:**
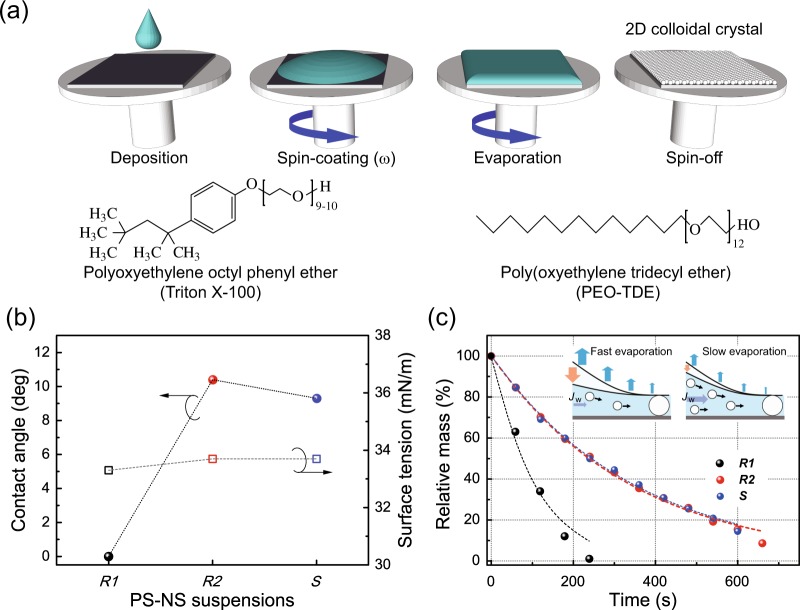
Coating process of PS NS colloidal suspensions and their surface wettabilities. (**a**) Upper: Schematic drawing of the spin-coating process for the fabrication of PS NS monolayers using colloidal suspensions. Lower: Molecular structures of the surfactants used, Triton X-100 and PEO-TDE. (**b**) The contact angles on piranha-cleaned silicon substrates and surface tension levels for drops of the three PS NS suspensions: *R1* with no surfactant, *R2* with the Triton X-100 surfactant, *S* with the PEO-TDE surfactant. (**c**) The solvent evaporation rates of the PS NS suspensions. The inset in (**c**) shows a schematic illustration of fast (left) and slow (right) evaporation rates of the solvent, causing short- and long-period *J*_w_s, respectively, during the spin-coating process.

The first challenge we address relates to the surface wettability of the PS NS suspension to understand the role of the surfactants used in the formation of the PS NS monolayer on the Si wafer substrate (Fig. 1a, see also Methods for details of the fabrication of the PS NS monolayer), which has a hydrophilic oxide surface having undergone a piranha cleaning step. In this study, we tested three types of spin-coating materials: (i) a PS NS aqueous colloidal suspension diluted with pure methanol (4:3 v/v) as a reference (hereafter, *R1*), (ii) a PS NS colloidal suspension diluted with methanol (4:3 v/v) mixed with Triton X-100 (1:400 v/v) as a comparative reference (hereafter, *R2*)^1,4,22^, and (iii) a PS NS colloidal suspension diluted with methanol (4:3 v/v) mixed with PEO-TDE (1:400 v/v) as a sample suspension (hereafter, *S*). To investigate the surface wettability of the suspension, which is a crucial factor to obtain a uniform monolayer of colloidal particles^1^, we initially studied the contact angles, surface tension levels, and viscosities of the suspensions, denoted here as *R1*, *R2*, and *S*.

Figure 1b shows three examples of contact angles observed for drops of *R1*, *R2*, and *S* onto the cleaned Si substrates. The measured contact angles were ca. ~0° (unmeasurable) for the *R1* suspension with no surfactant, ca. 10.4° for the *R2* suspension with Triton X-100, and 9.3° for the *S* suspension with PEO-TDE on the Si surfaces. From this comparison, it is clear that the *S* suspension exhibits a nearly identical contact angle to that of the *R2* suspension, slightly higher than that of the *R1* suspensions, whereas all of the suspensions show quite low contact angles on the hydrophilic Si wafers. It may thus be considered that the PEO-TDE surfactant molecules were coated onto the Si substrate and oriented normally to the Si surface; i.e., the hydroxyl polar head group (–OH) of PEO-TDE was anchored to the Si surface, while its alkyl chains protruded outwards, similar to the aromatic hydrocarbon lipophilic group in Triton X-100, both of which caused slight increases in the contact angles of the aqueous suspensions *R2* and *S*.

Figure 1b also shows the measured surface tension levels of the suspensions. The observed surface tensions were ca. 33.3 mN/m for *R1*, ca. 33.7 mN/m for *R2*, and 33.7 mN/m for *S*, which were nearly identical to each other. These lower surface tensions than that (72 mN/m) of DI water are mainly caused by the methanol mixed into the suspensions. Similar to the surface tensions, the observed viscosities of the suspensions were also nearly identical to each other, at ca. 1.88 cP for *R1*, ca. 1.91 cP for *R2*, and 1.90 cP for *S* (cf. ~1 cP for DI water). These observations indicate that the wettability of the *S* suspension with PEO-TDE is quite similar to that of the conventional *R2* suspension with Triton X-100.

Next, we observed the solvent evaporation rates of the suspensions used (Fig. 1c). As shown in the figure, it is clear that the *S* suspension with PEO-TDE exhibits a solvent evaporation rate nearly identical to that of *R2* with Triton X-100, which is higher than that of *R1* with no surfactant. This delayed evaporation rate of *S* with PEO-TDE provides long-period convective force in the convective particle flux (*J*_w_)^28,29^, which may lead to the formation of a uniform monolayer of colloidal PS NSs, as illustrated in the inset of Fig. 1c. Thus, according to these results, the PEO-TDE surfactant can be considered as a potential candidate for the uniform formation of 2D colloidal monolayer arrays of PS NSs instead of the conventional Triton X-100.

**Figure 2:**
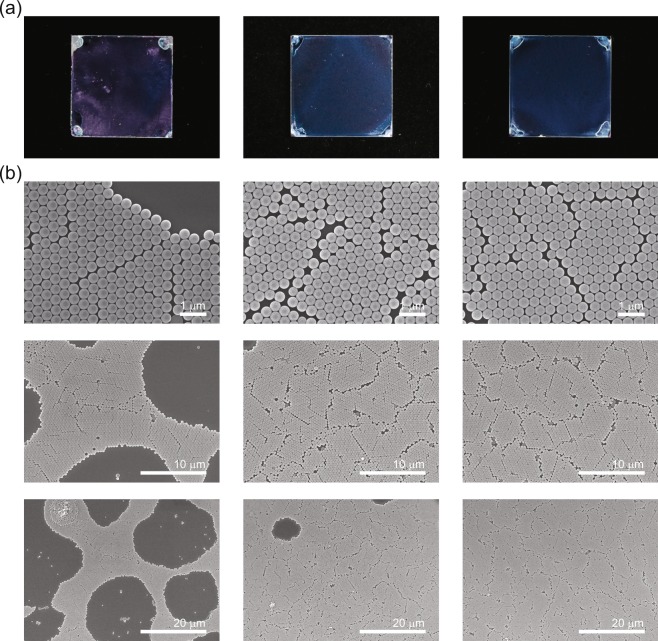
Macroscopic and microscopic morphologies and structures of the PS NS layers on Si substrates. Top-view macroscopic snapshots (**a**) and microscopic SEM images (**b**) at three different scales of PS NS layers fabricated on Si wafer substrates (2 × 2 cm^2^) with three different suspensions: (left) *R1* with no surfactant, (middle) *R2* with Triton X-100, and (right) *S* with the PEO-TDE surfactant. Note that the SEM images were taken from nearly the centre of each sample. Scale bars: 1 µm (top), 10 µm (middle), and 20 µm (bottom).

Next, in order to study the influence of the PEO-TDE surfactant on the quality of the spin-coated PS NS layers, we investigated the macroscopic and microscopic morphologies and structures of the PS NS layers fabricated on the Si wafer substrates. In Fig. 2a, for a direct comparison, we show macroscopic snapshot images of three spin-coated PS NS layers on piranha-cleaned 2 × 2 cm^2^ rectangular Si wafers using 100 μL of the *R1*, *R2*, and *S* suspensions. These images indicate that the *R2* and *S* suspensions provide uniform and homogeneous PS NS layers over the entire substrate area, except the four corners of each substrate. In contrast, the *R1* suspension with no surfactant gives rise to a relatively nonuniform layer. These improved uniformities of the PS NS layers with *R2* and *S* can be explained by their delayed solvent evaporation rates compared to that of *R1*, as shown in Fig. 1c. The delayed solvent evaporation rate due to the PEO-TDE surfactant in the *S* suspension leads to long-period *J*_w_s that deliver a sufficient amount of PS NSs homogeneously onto the substrate surface, similar to the conventional Triton X-100 surfactant in *R2*. Hence, it is clear that high macroscopic uniformity of the PS NS layer from the centre to the edge of the substrate can be achieved by the introduction of PEO-TDE instead of typical Triton X-100 into the PS NS suspension.

Next, the effect of the surfactant is also shown in the SEM images in Fig. 2b for the PS NS layers on the Si wafer substrates, where PS NS arrays reveal noticeable monolayer coverage of hexagonally close-packed PS NSs on the substrates. However, as shown in the figure, the SEM images of the PS NS layer fabricated using *R1* with no surfactant exhibit apparently large PS NS-free areas (voids), which significantly affect the monolayer coverage of PS NSs on the substrate. For the PS NS layers fabricated using *R2* and *S*, there are fewer PS NS-free voids on the substrates, but a few voids still appear (see below for further details). The reduced numbers of voids in the PS NS layers for *R2* and *S* mainly stem from the presence of the surfactants in the colloidal suspensions, which may improve the interparticle interactions and render the organization of the PS NSs more effective^22,27^. Thus, the introduction of the PEO-TDE surfactant instead of Triton X-100 may offer improved uniformity and monolayer coverage of the assembled PS NS layer.

### Defects and 2D ordering of PS NS layers

**Figure 3:**
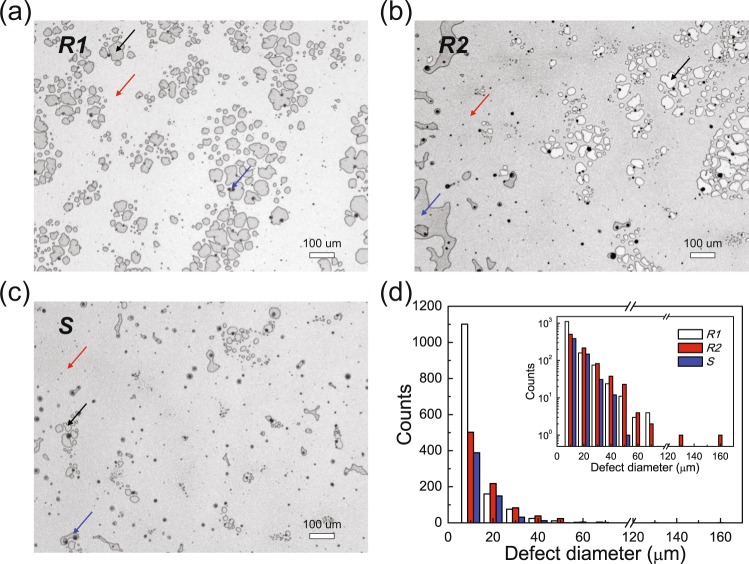
Non-contact 3D optical microscopic images of PS NS layers on Si substrates. (**a**) *R1* with no surfactant, (**b**) *R2* with Triton X-100, and (**c**) *S* with the PEO-TDE surfactant. Black, blue, and red arrows denote examples of voids, multilayers, and monolayers, respectively. (**d**) Defect distributions of the three PS NS layers as measured by a 3D optical surface profiler. The inset shows the plot in a semi-logarithmic scale.

Our investigation also focused on the effect of the surfactant on the coverage of the assembled PS NS layer. We investigated the microscopic surface morphologies of the PS NS layers using a non-contact 3D optical surface profiler. In Fig. 3, we compare representative microscopic surface images of the three PS NS layers on the Si wafers for *R1*, *R2*, and *S*. The microscopic image in Fig. 3a shows that the surface of the PS NS layers without a surfactant appears to be rough (RMS roughness of ~93 nm) with PS monolayer coverage of ~83% due to many large defects, such as voids and multilayers. For comparison, Fig. 3b shows that the surface of the PS NS layers with Triton X-100 presents a RMS roughness of ~88 nm and monolayer coverage of ~82%, similar to those of the PS NS layer without a surfactant but also exhibiting some changes in defects, *i*.*e*., reduced voids but increased multilayers. These changes in the defects can also be explained by the delayed solvent evaporation rate of *R2* with Triton X-100 compared to the rate of *R1* with no surfactant, as noted above. The long-period hydrodynamic flux *J*_w_s due to the Triton X-100 surfactant in *R2* deliver a sufficient amount of PS NSs to the substrate surface regions from the suspension flows, resulting in reduced voids and increased multilayers, as illustrated in the inset of Fig. 1c.

In contrast, interestingly, as shown in Fig. 3c, it was found that the surface of the PS NS layers with PEO-TDE presents a relatively smooth monolayer morphology with greatly reduced voids and multilayers. The estimated monolayer coverage of the assembled PS NS layer with PEO-TDE is improved significantly to 95% and the RMS roughness is also decreased to ~47 nm, both of which are much better than those without (*R1*) and with Triton X-100 (*R2*). Also note that the high monolayer coverage of up to 95% for the PS NS layers with PEO-TDE represents one of the highest levels of reported coverage realized by a spin-coating method^22–24^. For a detailed comparison, the distributions of the defects for the three PS NS layers were also estimated. These results are plotted in Fig. 3d. From the defect distributions, it is clear that the large defects over 10 μm in the PS NS layer with PEO-TDE were remarkably reduced in compared to those in the reference PS NS layers without (*R1*) and with Triton X-100 (*R2*). These outcomes demonstrate that high microscopic uniformity of the PS NS layer on the Si substrate can be readily achieved by the introduction of the PEO-TDE surfactant into the PS NS suspension.

**Figure 4:**
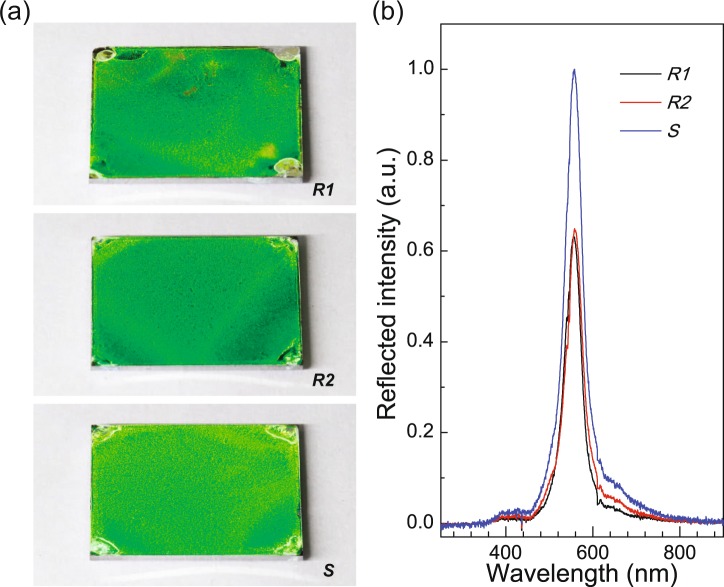
Optical reflection from PS NS layers assembled on Si substrates. Photographs of light reflection from PS NS layers without a surfactant and with different surfactants (**a**), and their reflection spectra (**b**) at the Littrow configuration with an incident angle of 50°. *R1*: PS NS layer with no surfactant, *R2:* PS NS layer with Triton X-100, *S*: PS NS layer with PEO-TDE.

Next, we describe the long-range ordering of the PS NS monolayers on the Si substrates by observing the optical reflection from the layers, as commonly observed in conventional 2D/3D photonic crystals (Fig. 4)^30^. Here, the observation angle of the sample surface was set to 50° with Littrow geometry, i.e., backscattering geometry^31^, resulting in the diffraction of light at a wavelength of about 560 nm (greenish yellow), as shown in Fig. 4a. Interestingly, in contrast to those of the reference layers without (*R1*) and with Triton X-100 (*R2*), the diffracted light from the PS NS monolayer with PEO-TDE (*S*) was highly homogeneous and uniform over the entire surface of the substrate (except for only the four corners of the square substrate). To confirm this observation, we also measured the relative intensity spectra of diffracted light from the surfaces (Fig. 4b). As shown in the figure, the highly reflected light at around ~560 nm is mainly caused in each case by the strong diffraction of incident light caused by the close packed array of the PS NS monolayers^30–32^. The reflected light intensity at 560 nm from the PS NS monolayer with PEO-TDE (*S*, FWHM ~ 47 nm) is ~1.57 times higher than that without surfactant (*R1*, FWHM ~ 46 nm), while the reflected intensity from the PS NS monolayer with Triton X-100 (*R2*, FWHM ~ 45 nm) is nearly the same as that without surfactant (*R1*). This observation provides strong evidence that the 2D long-range crystalline ordering of the PS NS monolayers with the PEO-TDE surfactant is greatly improved compared to those without and with the conventional Triton X-100.

### Effects of surfactants on self-assembled monolayers of PS NPs

We now turn our attention to the effects of surfactants on the mololayer formation of PS NPs. The high monolayer coverage and few defects with the high 2D ordering for the PS NS layer with PEO-TDE indicate that during the spin-coating process, the PS NSs were dispersed homogeneously in the suspension layer on the substrate with appropriately delayed solvent evaporation due to the PEO-TDE surfactant. However, such significantly improved uniformity of the PS NS layer with PEO-TDE, in comparison with those with Triton X-100 (*R2*), cannot be explained solely by the delayed solvent evaporation rate, as the rate of *S* is nearly identical to that of *R2*, as shown in Fig. 1c. Hence, to identify any differences in the effects of the surfactant on the monolayer formation of the PS NSs, we turn our attention to the changes in the viscosity and surface tension levels of the suspensions during the spin-coating process. During this process, evaporation of the solvent takes place from the coated fluid layer of the suspension; thus, the concentration of the surfactant in the fluid layer increases further as the spinning time progresses. Therefore, in order to understand the coating conditions during the spin-coating process, we investigated the surface tension and viscosity of the suspensions as a function of the concentration of the surfactant. For this observation, we prepared PS NS suspensions by adding a surfactant into the suspension but without methanol to dilute the suspension, because methanol mixed in the suspension evaporates initially at the early stage of the spin-coating process.

**Figure 5:**
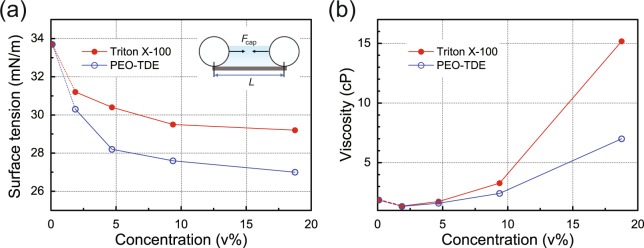
Surfactant concentration dependent characteristics of PS NS suspensions. The surface tension (**a**) and viscosity (**b**) levels of PS NS suspensions as a function of the concentration of the surfactants, Triton X-100 and PEO-TDE. The inset in (**a**) shows a schematic illustration of the capillary force *F*_cap_ with interparticle distance *L*.

Figure 5 shows the surface tension and viscosity outcomes of the PS NS suspensions for various surfactant concentrations. The surface tension results in Fig. 5a indicate that as the concentration of the surfactant increases, the surface tension begins to decrease from 33.7 to 29.2 mN/m for Triton X-100 and from 33.7 to 27.0 mN/m for PEO-TDE. Accordingly, the changes in the surface tensions are in the approximate range of 13~20%. Therefore, during the spin-coating process, the evaporation of the water solvent in the suspensions induces small decreases in the surface tensions *γ* of the suspensions, which may have little influence on the formation of the PS NS assemblies due to the slightly reduced capillary force ${F}_{{\rm{c}}{\rm{a}}{\rm{p}}}\,({{F}}_{{\rm{c}}{\rm{a}}{\rm{p}}}\propto \gamma )$ between the PS NSs^27,33–35^, as illustrated in the inset of Fig. 5a.

On the other hand, the viscosities in Fig. 5b show that as the concentration of the surfactant increases, the viscosity of the suspension with Triton X-100 increases very sharply (more than ~15.2 cP) because of its strong gel forming tendency with water (at near ~50 v%)^26^, in contrast to the relatively small increase in the viscosity of the suspension with PEO-TDE. Thus, during the spin-coating process, the evaporation of the water solvent in the suspensions induces a large increase in the viscosity (>15 cP) of the suspension with Triton X-100, possibly leading to severe nonuniformity and defects in the PS NS layers^27^, as shown in Fig. 3b. In contrast, the relatively small increase in the viscosity of the suspension with PEO-TDE during the spin-coating process disturbs the formation of uniform fluid layer of the suspension on the substrate less, resulting in less severe defects in the uniform PS NS monolayer. Therefore, it is clearly demonstrated that the significantly improved uniformity of the PS NS monolayer with PEO-TDE, as shown in Fig. 3c, can be explained by the relatively low viscosity of the PS NS suspension even at a high concentration of PEO-TDE. Thus, the PS NS suspension with PEO-TDE shows considerable promise for the construction of reliable, uniform, and highly ordered 2D colloidal crystals.

**Figure 6:**
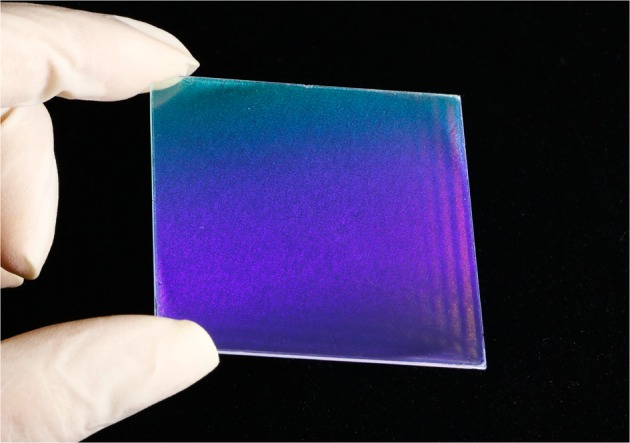
Large area spin-coated PS NS monolayer on glass substrate using a PS NS suspension with PEO-TED. Photograph of a spin-coated large-scale PS NS monolayer on a large glass substrate (5 × 5 cm^2^) using a suspension (PS NS diameter = 500 nm) with the PEO-TDE surfactant.

Finally, encouraged by the above impressive results of the spin-coated PS NS monolayer with PEO-TDE, we also fabricated a large-scale PS NS monolayer on a 5 cm × 5 cm glass substrate using a suspension containing differently sized PS NSs (diameter = 500 nm) to assess the processability of the PS NS suspensions with PEO-TDE. A photographic image of a large-scale PS NS monolayer fabricated on a large glass substrate is shown in Fig. 6. The figure clearly shows that with the spin-coating method together with the PEO-TDE surfactant, a fairly homogeneous and uniform PS NS monolayer was successfully deposited over the entire glass substrate, despite a few insignificant defects on the surface. Moreover, the low variation in the reflected light intensity over the entire surface area implies small variations in the monolayer of PS NSs with reduced defects, providing evidence of the suitability of the PEO-TDE surfactant even for large-scale fabrication with PS NSs of various sizes.

## Conclusions

In summary, we have introduced PEO-TDE as a surfactant for the spin-coating of PS NS monolayers on Si wafers and glass substrates under ambient laboratory conditions, as opposed to the conventional highly toxic Triton X-100 surfactant. In comparison with Triton X-100, it is clear that PEO-TDE offers optimal surfactant properties for spin-coating applications of PS NSs. We have verified the excellent wettability of the PS NS suspension with PEO-TDE, caused by its low viscosity together with low surface tension and a low contact angle on the Si wafer substrate, imparting superior coverage and uniformity of the PS NS monolayer compared to the suspension with Triton X-100. Moreover, the slow evaporation rate of the suspension with PEO-TDE in conjunction with the low viscosity of the suspension even at a high concentration of PEO-TDE is highly desirable for spin-coating applications. On the basis of these outstanding properties of the suspension with PEO-TDE, we successfully demonstrate high uniformity and monolayer coverage (~95%) of a PS NS monolayer on a Si substrate. Furthermore, we demonstrated a large-scale PS NS monolayer with a differently sized diameter on a 5 cm × 5 cm glass substrate using the PEO-TDE-controlled spin-coating method. These achievements demonstrate that the introduction of PEO-TDE into the PS NS suspension offers great potential for a simple and low-cost spin-coating approach to produce highly uniform 2D colloidal crystals over a large-scale surface. Together with its simple processability, the high uniformity and monolayer coverage of the PS NS monolayer with the PEO-TDE surfactant provide a new platform for the development of surfactant-assisted deposition processes for shadow nanosphere lithography, 2D optical devices, solar cells, and bio/chemical sensors and chips.

## Methods

### Materials and preparation of substrates

All reagents were purchased from commercial sources and were used without further purification. Aqueous suspensions of PS NSs (Polybead®Microspheres, diameter: 0.35 μm or 0.50 μm, 2.5% w/v) were purchased from Polysciences, Inc. Nonionic surfactants, Triton X-100 ((C_2_H_4_O)_n_C_14_H_22_O, n = 9,10) and PEO-TDE (C_13_H_27_(OCH_2_CH_2_)_n_OH, n = 12), were purchased from Sigma-Aldrich.

The PS NS suspensions from the manufacturer were diluted by adding methanol at a ratio of 4:3 v/v after the surfactant of PEO-TDE or Triton X-100 was pre-dissolved in methanol at a concentration of 1:400 v/v^1,4,22^. Prior to use, the PS NS suspensions were sonicated for 1 h for complete dispersion of the PS NSs in the suspension. The viscosity of the PS NS suspension was measured using a viscometer (RVDV II+, Brookfield Inc.).

The fabrication of the PS NS layers followed a well-established process (see Fig. 1a). A 2 × 2 cm^2^ rectangular polished *n*-type Si (100) substrate with a 300-nm-thick SiO_2_ layer (or a 5 × 5 cm^2^ glass substrate) was used as the substrate and was cleaned in an ultrasonic bath successively in acetone, ethanol and deionised (DI) water for 30 min, 30 min, and 30 min, respectively, after which the substrate was dried. Prior to use, for a surface treatment to form a hydrophilic surface, the substrate was immersed into an oxidant solution containing a mixture of H_2_SO_4_ (97%) and H_2_O_2_ (35%) with a volume ratio of 4 to 1 (v/v) for 1 h, followed by a rinse with DI water. Subsequently, to render the surface hydrophilic, the substrate was treated by immersing it into a solution of H_2_O: NH_4_OH: 30% H_2_O_2_ (5: 1: 1 v/v/v) at 80 °C, after which the substrate was washed again with DI water and dried.

### Fabrication and characterization of PS NS monolayers

After the above substrate treatment, the PS NS monolayer was fabricated on the substrate by a spin-coating process. The volume of the suspension for spin-coating varies with the size of the substrate, e.g., 100 μl for a 2 × 2 cm^2^ substrate. While dropping 100 μL of the suspension onto the substrate mounted on the spin coater, a three-step spin-coating process was used for the self-assembly of the PS NSs to achieve a highly oriented and close-packed monolayer as an optimal condition for spin coating. The first step ran at 300 rpm for 5 s after acceleration for 20 s, the second step was done at 700 rpm for 5 s after acceleration for 60 s, and the third step operated at 1200 rpm for 5 s after acceleration for 20 s.

PS NS suspensions and their layers were evaluated with various characterization techniques. The contact angles and surface tensions of the PS NS suspensions were measured by the static sessile drop method using a contact angle meter (Attension Theta, KSV Instruments Ltd.). The microscopic morphologies and structures of the fabricated PS NS layers were investigated using a field emission scanning electron microscope (SEM, Model JSM-6700F, JEOL Co.) and a non-contact 3D optical surface profiler (NV-2400, Nanosystem Co. Ltd.). The coverage of the PS NS monolayer was calculated by the direct counting of the covered area of PS NSs on the substrate with the image analysis software ImageJ (National Institutes of Health, USA). Optical reflection spectra from the PS NS layers were measured using a spectrometer (HR4000, Ocean Optics).
